# Phylogeny of *Cirsium* spp. in North America: Host Specificity Does Not Follow Phylogeny

**DOI:** 10.3390/plants1020061

**Published:** 2012-10-24

**Authors:** Tracey A. Bodo Slotta, David P. Horvath, Michael E. Foley

**Affiliations:** 1 Biology Department, American University, 4400 Massachusetts Ave. NW, Washington, DC 20016, USA; Email: tracey.slotta@gmail.com; 2 Sunflower and Plant Biology Research Unit, USDA-Agricultural Research Service, 1605 Albrecht Blvd, Fargo, ND 58102, USA; Email: david.horvath@ars.usda.gov

**Keywords:** biological control, Canada thistle, *Cirsium*, *Cirsium arvense*, phylogeny, thistles, weed

## Abstract

Weedy invasive *Cirsium* spp. are widespread in temperate regions of North America and some of their biological control agents have attacked native *Cirsium* spp. A phylogenetic tree was developed from DNA sequences for the internal transcribed spacer and external transcribed spacer regions from native and non-native Great Plains *Cirsium* spp. and other thistles to determine if host specificity follows phylogeny. The monophyly of *Cirsium* spp. and *Carduus* within the tribe Cardinae was confirmed with native North American and European lineages of the *Cirsium* spp. examined. We did not detect interspecific hybridization between the introduced invasive and the native North American *Cirsium* spp. Selected host-biological control agent interactions were mapped onto the phylogenic tree derived by maximum likelihood analysis to examine the co-occurrence of known hosts with biological control agents. Within *Cirsium*-Cardueae, the insect biological control agents do not associate with host phylogenetic lines. Thus, more comprehensive testing of species in host-specificity trials, rather than relying on a single representative of a given clade may be necessary; because the assumption that host-specificity follows phylogeny does not necessarily hold. Since the assumption does not always hold, it will also be important to evaluate ecological factors to provide better cues for host specificity.

## 1. Introduction

The invasion history, genetic diversity associated with founding populations, and evolutionary relationships to proximal native species should be considered in developing biological control management strategies for weeds [1,2]; gaps in such knowledge have led to failures in biological control [3]. Indeed, assessment of genetic diversity in invasive plant populations can assist in predicting the effectiveness and longevity of herbicidal, biological, and other control measures [4,5,6,7].

Canada thistle (*Cirsium* arvense (L.) Scop.) is one of the world’s most serious weeds and is a highly invasive plant in temperate regions of North America [8,9]. The introduction of Canada thistle to North America is suspected to result from contaminated goods shipped from Europe [10]. In 1795, Vermont was the first state to identify it as a noxious weed [11]. Canada thistle is now classified as a noxious weed by 49 states/provincial governments [12] because it causes economic loss through reduced crop yield, deterred grazing, and habitat loss in natural areas [9,13]. Auxin-type herbicides provide some control of Canada thistle; however, the most effective control is obtained by integration of chemical, mechanical, cultural, and biological control methods [8,14]. For example, multiple applications of the native bacterial biological control agent *Pseudomonas syringae* pv. *tagetis* in conjunction with other control measures were necessary to produce infection and sufficient damage to control growth and seed production of Canada thistle [15].

Several insects have been introduced to North America as biological control agents for non-native thistles (*Cirsium arvense*, *Cirsium vulgare*, and *Carduus nutans* (=*Carduus thoemeri*). The root and stem weevil *Hadroplontus litura* (=*Ceutorhynchus litura*) was released in North America in 1971 specifically for *C. arvense* control, but this agent has had little or no impact on *C. arvense* populations [8,16]. The weevils *Rhinocyllis conicus* and *Larinus planus* were introduced with some success for control of *C. nutans* (musk thistle). However, these insect species have non-target effects. For example, *L. planus* attacks *C. arvense* and the native *Cirsium undulatum*, as well as other native North American species [17,18,19]. Unfortunately, the risks associated with the release of biological control agents are not typically fully evaluated with regard to the native flora [18]; the focus is generally non-target effects on economically important plants like crops and forages [8].

Kelch and Baldwin [20] examined genetic diversity and ecological variation of North American *Cirsium* to determine the timing of New World thistle diversification, particularly those of the California Floristic Province. The phylogenetic estimates used maximum likelihood analysis of external transcribed spacer (ETS) and the internal transcribed spacer (ITS) of nuclear ribosomal DNA (nrDNA) sequence. Single origins were indicated for old world *Cirsium*, New World *Cirsium*, and representatives from the California Floristic Province. They indicated further sampling within the Cardueae was required to substantiate that *Cirsium* and *Carduus* are monotypic genera; these species were attributed to a single genus in the past. Inclusion of additional Great Plains representatives of these species would assist in determining relationships among taxa that are not well-delimited morphologically and co-occur in areas where *C. arvense* infestations are greatest.

We examined genetic variation and population structure within and between *C. arvense* populations to develop a greater understanding of the biology and reproductive mechanism [21,22]. The level of diversity within populations was greater than expected for a clonally reproducing perennial, indicating high level of outcrossing between populations in North America. Here, we evaluate the relationship of invasive and native North American thistles. The interaction (e.g., hybridization, introgression) of non-native invasive and native endemic thistle species in the Great Plains of North America was also evaluated to identify any increased potential of non-target effects with biological control agents. The occurrences of some known biological control agents were mapped onto the resulting phylogeny to investigate the patterns of host specificity and preference.

## 2. Results

Direct sequencing of polymerase chain reaction (PCR) products was successful for all samples (Table 1). Pairwise sequence similarity of the ETS, ITS1, and ITS2 regions ranged from 100% within *Cirsium flodmanii *(populations SD7, TS, and 905) for the ingroup of *Cirsium* taxa to 51.2% between *Centaurea rigida* and *Tagetes* spp. when considering all species. Within *C. arvense*, the greatest sequence similarity was for *C. flodmanii* representatives (100%) and the least similarity was for *Cirsium monocephalum* (91.3%). In species with multiple populations, average pairwise similarity was *C. arvense* 98.3%, *C. flodmanii* 99.9%, *Cirsium muticum* 99.6%, *C. pitcheri* 99.4%, *C. undulatum* 99.4%, and *C. vulgare* 99.5%.

**Table 1:** Species for phylogenetic analysis are indicated. Sequences generated for the current study are indicated by the population identification numbers with species (e.g., arvense IN1.1), corresponding collection information (Sources of TBS and MF (authors) and collaborator (J. Fant), or voucher number). Voucher specimens were deposited at the U.S. National Arboretum (NA) herbarium and the accession number is provided. Published sequences included in the analysis are indicated by the accession number of GenBank.

*Genus*	*Species*	Source	Voucher at NA	ITS	ETS
*Cirsium*	*arvense IN1.1*	TBS2004-13	48765	JX867618	JX867646
*Cirsium*	*arvense Itasca 1*	TBS2004-70	48793	JX867619	JX867647
*Cirsium*	*arvense Itasca 6*	TBS2004-71	48792	JX867620	JX867648
*Cirsium*	*arvense MN3*	TBS2004-33	48790	JX867621	JX867649
*Cirsium*	*arvense ND25s8*	TBS2004-72	48842	JX867622	JX867650
*Cirsium*	*arvense ND26s35*	TBS2004-58	48834	JX867623	JX867651
*Cirsium*	*arvense TS*	TBS2004-30	48817	JX867624	JX867652
*Cirsium*	*canescens SD7*	TBS	----	JX867625	JX867653
*Cirsium*	*canovirens*	TBS2005-36	48883	JX867626	JX867654
*Cirsium*	*flodmanii 64*	TBS2004-64	48867	JX867627	JX867655
*Cirsium*	*flodmanii 905*	TBS2004-62	48861	JX867628	JX867656
*Cirsium*	*flodmanii SD7*	TBS2005-25	48870	JX867629	JX867657
*Cirsium*	*flodmanii TS*	TBS2004-60	48872	JX867630	JX867658
*Cirsium*	*foliosum*	TBS2005-33	48878	JX867631	JX867659
*Cirsium*	*hillii*	Jeremie Fant, Chicago Bot Gard		JX867632	JX867660
*Cirsium*	*muticum*	TBS2005-17	48873	JX867633	JX867661
*Cirsium*	*pitcheri*	Jeremie Fant, Chicago Bot Gard		JX867634	JX867662
*Cirsium*	*undulatum 903.1*	TBS2004-59	48877	JX867635	JX867663
*Cirsium*	*undulatum 904.1*	TBS2004-59	48877	JX867636	JX867664
*Cirsium*	*undulatum SD*	TBS sn. 27 July 2005	48876	JX867637	JX867665
*Cirsium*	*vulgare 1.1*	MF2	48794	JX867638	JX867666
*Cirsium*	*vulgare 2.1*	MF2	48794	JX867639	JX867667
*Cirsium*	*vulgare SD7*	TBS2005-26	48812	JX867640	JX867668
*Cirsium*	*andersonii*	GenBank		AF443683	AF443735
*Cirsium*	*andrewsii*	GenBank		AF443684	AF443736
*Cirsium*	*arvense clone 1*	GenBank		AF443680	AF443734
*Cirsium*	*arvense clone 2*	GenBank		AF443681	AF443734
*Cirsium*	*arvense clone 3*	GenBank		AF443682	AF443734
*Cirsium*	*brevistylum*	GenBank		AF443685	AF443737
*Cirsium*	*calcareum*	GenBank		AF443687	AF443739
*Cirsium*	*canovirens*	GenBank		AF443688	AF443740
*Cirsium*	*canum*	GenBank		AF443689	AF443741
*Cirsium*	*congdonii*	GenBank		AF443690	AF443742
*Cirsium*	*cymosum*	GenBank		AF443691	AF443743
*Cirsium*	*discolor*	GenBank		AF443692	AF443744
*Cirsium*	*douglasii*	GenBank		AF443686	AF443738
*Cirsium*	*eatonii*	GenBank		AF443694	AF443746
*Cirsium*	*edule*	GenBank		AF443711	AF443763
*Cirsium*	*ehrenbergii*	GenBank		AF443726	AF443778
*Cirsium*	*faucium*	GenBank		AF443725	AF443777
*Cirsium*	*fontinale var. obispoense*	GenBank		AF443696	AF443748
*Cirsium*	*henryi*	GenBank		AF443697	AF443749
*Cirsium*	*hydrophilum*	GenBank		AF443698	AF443750
*Cirsium*	*jorullense*	GenBank		AF443699	AF443751
*Cirsium*	*lineare*	GenBank		AF443727	AF443779
*Cirsium*	*mohavense*	GenBank		AF443700	AF443752
*Cirsium*	*monocephalum*	GenBank		AF443701	AF443753
*Cirsium*	*monspessulanum*	GenBank		AF443717	AF443769
*Cirsium*	*muticum*	GenBank		AF443722	AF443774
*Cirsium*	*neomexicanum*	GenBank		AF443718	AF443770
*Cirsium*	*occidentale var. venustum*	GenBank		AF443703	AF443755
*Cirsium*	*occidentale*	GenBank		AF443702	AF443754
*Cirsium*	*palustre*	GenBank		AF443704	AF443756
*Cirsium*	*pitcheri*	GenBank		AF443705	AF443757
*Cirsium*	*quercetorum*	GenBank		AF443706	AF443758
*Cirsium*	*remotifolium*	GenBank		AF443707	AF443759
*Cirsium*	*rhaphilepis*	GenBank		AF443708	AF443760
*Cirsium*	*rhothophilum*	GenBank		AF443709	AF443761
*Cirsium*	*rydbergii*	GenBank		AF443710	AF443762
*Cirsium*	*scariosum*	GenBank		AF443693	AF443745
*Cirsium*	*spinosissimum*	GenBank		AF443720	AF443772
*Cirsium*	*subniveum*	GenBank		AF443712	AF443764
*Cirsium*	*tioganum*	GenBank		AF443721	AF443773
*Cirsium*	*tymphaeum*	GenBank		AF443723	AF443775
*Cirsium*	*utahense*	GenBank		AF443713	AF443765
*Cirsium*	*velatum*	GenBank		AF443714	AF443766
*Cirsium*	*vulgare clone 1*	GenBank		AF443715	AF443767
*Cirsium*	*vulgare clone 2*	GenBank		AF443716	AF443738
*Cirsium*	*wheeleri*	GenBank		AF443719	AF443771
*Carduus*	*acanthoides*	MF3	48795	JX867643	JX867669
*Carduus*	*nutans*	GenBank		AF443678	AF443730
*Carduus*	*nutans*	TBS2005-14	48801	JX867642	JX867670
*Carduus*	*tenuiflorus*	GenBank		AF44679	AF4433731
*Carthamus*	*oxyacanthus*	GenBank		AJ867986-7	AJ867985
*Centaurea*	*rigidi*	GenBank		AJ867989	AJ867988
*Cynara*	*scolymus*	TBS	greenhouse grown	JX867643	JX867671
*Helianthus*	*anuus*	TBS	greenhouse grown	JX867644	Not sequenced
*Jurinea*	*narynensi*	GenBank		AJ868001-2	AJ868000
*Onopordum*	*acaulon*	GenBank		AF443676	AF443728
*Onopordum*	*illyricum*	GenBank		AY78046	AF4433729
*Saussurea*	*riederi*	GenBank		AJ868070-1	AJ868069
*Tagetes*	spp.	TBS	greenhouse grown	JX867645	JX867672

Eighty-nine sequences representing 59 species were analyzed for ITS1, ITS2, and ETS for the total combined analysis; the 5.8S nrDNA region was excluded due to missing data for several taxa in GenBank (Table 1). Partition homogeneity indicated (*p* = 0.01) the ITS and ETS data sets were not incongruent. When considering all samples, 414 out of 1,082 base pair alignments analyzed were parsimony informative and 154 were parsimony informative within the ingroup of *Cirsium*. Thirty-seven most parsimonious trees arose with a tree length of 1,574 steps in a heuristic search using tree bisection-reconnection (TBR) branch-swapping with random addition of 1,000 bootstrap replicates. Bootstrap analysis conducted using the above parameters indicated high support (>75%) for 22 clades and moderate support (50–75%) for an additional 13 clades. Most notably, there was high support for the genus *Cirsium* (82%) and *Carduus* (100%), as well as species clades: *C. arvense* (95%), *C. vulgare* (97%), *C. flodmanii* (98%), and *C. undulatum* (100%). Bootstrap support values greater than 50% were not obtained for unique North American or European *Cirsium* clades. Taxa did not form robust clades based upon geographical regions within North American endemic *Cirsium*, except for a clade of the endemic California taxa.

**Figure 1:**
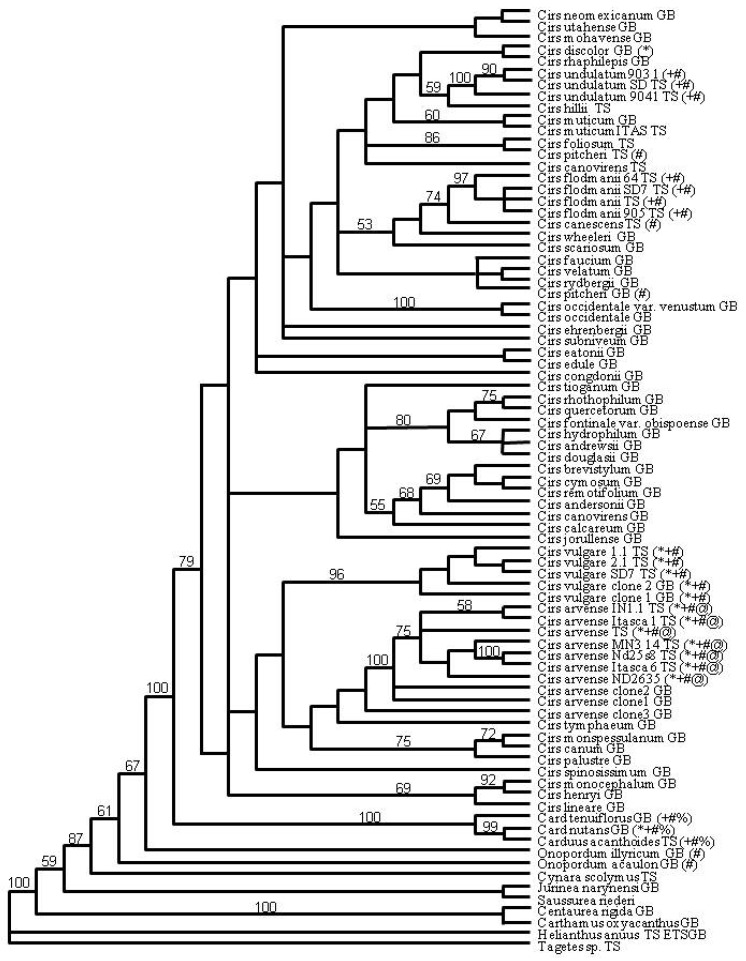
Maximum likelihood analysis of ITS1, ITS2, and ETS. Biological control agents are labeled as follows *Trichosirocalus horridus *(*), *Larinus planus* (+), *Rhinocyllis conicus* (#), *Puccinia carduorus* (%), and *Pseudomonas syringe* (@). Cirs = *Cirsium*, Card = *Carduus*, TS = sequence generated here, GB = sequence available on GenBank. Bootstrap values (1,000 iterations) are indicated when support was greater than 50%.

A greater resolution of species relationships was obtained using maximum likelihood analysis (Figure 1). Separate clades were obtained for the North American and the European *Cirsium* species in addition to species clades obtained from the bootstrap analyses. Known host-biological control agent interactions were mapped onto the phylogenic tree (Figure 1). Within *Cirsium*-Cardueae, the majority of biological control agents do not associate with the phylogenetic lines.

## 3. Discussion

*Cirsium* and *Carduus* are monophyletic genera based on our analysis, supporting Kelch and Baldwin [20] and Garcia-Jacas *et al*. [23]. The genera are morphologically distinct with the plumouse pappus of *Cirsium* and the generally winged stems of *Carduus* [24]. Tribal relationships resolved in our investigation also support those of Garcia-Jacas *et al*. [23]. The Cardinae subtribe composed of the *Carduus* group, *Onopordum*, *Cynara*, *Jurinea*, and *Sassurea* did not resolve with >50% bootstrap support and is paraphyletic with *Centaurea*, consistent with analysis of ITS and matK [23].

Fine scale analyses within *Cirsium* did not resolve phylogeographic relationships; for example, the Great Plains species do not form a clade. A single origin is indicated for the North American taxa separating these from the European species, but bootstrap support was weak (<50%). This single origin is in general agreement with Kelch and Badwin [20]. The clade (80% bootstrap) of endemic California species is the only group reflecting geographical distribution; however, other species with broader distributions were not included in this clade. These narrow endemics are most likely of recent origin derived from taxa found west of the Rocky Mountains [20].

Representatives included from the Northern Great Plains (east of the Rocky Mountains, west of the Great Lakes and north of Nebraska) formed in clades based on species identity when multiple populations were sampled, but did not segregate as a geographic group. A loosely grouped (e.g., short branch lengths and lack of bootstrap support) set of *Cirsium canescens*, *Cirsium canovirens*, *Cirsium discolor*, *C. flodmanii*, *Cirsium foliosum*, *Cirsium hillii*, *Cirsium pitcheri*, *Cirsium scariosum*, *C. undulatum*, and *Cirsium wheeleri* resolved as paraphyletic with several Mexican taxa [25]. The majority of these taxa are thought to have originated in a species complex in the mountainous, western regions of North America [26].

Chromosomal numbers observed for *Cirsium brevifolium* (2n = 22), *C. canovirens* (2n = 34), *C. flodmanii* (2n = 22), *C. pitcheri* (2n = 34), *C. undulatum* var. *tracyi* (syn. *C. tracyi* 2n = 24), *C. undulatum* (2n = 26), and *C. wheeleri* (2n = 28) led to their placement in the series Undulata with a basal chromosome number of 2n = 34 and subsequent reduction during species diversification and expansion [26]. *Cirsium altissimum* (2n = 18), *C. discolor* (2n = 20), and *C. muticum* (2n = 20) were placed in separate series (Altissima) based on morphological characters and thought to be derived within an eastern, plains to rolling hills complex of taxa. Several distinctions between *C. altissimum*, *C. brevifolium*, and *C. flodmanii* to others in the Undulata series include the lack of mucilage on wet achenes and the presence of a yellow apical band on achenes. Considering the distribution of *C. flodmanii* and *C. pitcheri*, with no locations west of the Rocky Mountains, these species may be derived from an eastern complex of taxa that moved westward, as reflected in the lack of a well-supported clade of the Undulata series. Separation of these taxa into the defined series is not supported as paraphyly of the biogeographical groups. No gene flow between these species was indicated by the ITS and ETS sequences analyzed. Thus, we conclude that these species remain genetically distinct. 

Morphological similarities and distributional overlap do not correspond to the phylogeny as *C. canescens*, *C. discolor*, *C. flodmanii*, and *C. undulatum* resolve as moderately to strongly supported unique species in separate clades based on the molecular analyses. These species are difficult to distinguish morphologically based on a gradation of leaf and stem pubescence, depth of leaf sinuses, and flower head shape [27]. Additionally, habitat preference and distribution also delimit these taxa. *C. discolor* is distributed further east (western Dakotas to the Atlantic) than the other taxa, with *C. flodmanii* (Michigan to Idaho) and *C. canescens* (Great Plains) in prairie habitats, and *C. undulatum* (Indiana, Texas to the Pacific) in dry grasslands [28].

Concerted evolution has been sufficient in the introduced and North American endemic species to homogenize ribosomal repeat region. Conspecifics formed independent clades with North American (*C. muticum*, *C. flodmanii*, and *C. undulatum*) and worldwide (*C. arvense*, *C. vulgare*) distributions. Concerted evolution of the nrDNA has occurred with the North American endemic species since their separation from Eurasian taxa during the Late Miocene (12 million years ago) [29]. The relatively recent introduction of the Eurasian *C. arvense* and *C. vulgare* (<300 years ago) to North America, in conjunction with the relatively low sequence divergence and high degree of concerted evolution of the nrDNA, supports continued gene flow within these species across North America or lack of lineage sorting. Strong support for clades consisting of representatives across the range for *C. arvense* and *C. vulgare* indicate a large source of genetic diversity in their ranges and potentially multiple introduction events consistent with Guggisberg *et al*. [10], at least for *C. arvense*.

The insect biological control agents do not follow the phylogenic relationships of hosts as judged by specificity of biological control options for *Cirsium arvense*, *C. vulgare*, and *C. nutans* (Figure 1). The weevil *L. planus* feeds upon *C. arvense* and *C. palustre* and other *Cirsium*, *Carduus*, *Galactities*, and *Serratula* species over its native range from Europe to North Africa [30]. *L. planus* is now found throughout the Great Plains of North America in areas with heavy infestations of invasive thistles. Its larvae develop within flower heads destroying florets leading to up to 95% suppression of seed production in *C. arvense* and *Carduus*, but also in the native thistles such as *C. undulatum* var. *tracyi* and *C. flodmanii* [17]. There is no correlation between host phylogenetic relationships and non-target effects as determined by phylogenetic mapping of thistle species affected by *L. planus* (Figure 1). Basically, *L. planus* is opportunistically feeding upon native species when there is an insufficient source of the targeted hosts like *C. arvense*.

*Rhinocyllus conicus* attacks seed heads of *Carduus* spp., *Cirsium* spp., and *Silybum marianum* within its native range in Europe [31]. Host-plant specificity tests in Europe for feeding, ovipositing, and better larval performance on *C. nutans* than on the *Cirsium* spp. influenced its selection as a biological control agent for *C. nutans* in North America [32]. Its introduction had mixed results [33,34]. Although the most efficient ovipositing and larval development in seeds heads occurred for *C. nutans*, *C. arvense*, and *C. vulgare* in North America, ovipositing was also discovered to occur in the native North American thistle species *C. canescens*, *C. centaureae*, *C. flodmanii*, *C. pitcheri*, *Cirsium pulchellum*, and *C. undulatum* [34]. The native thistles have greater pubescence and are genetically distant from the invasive species (Figure 1), yet non-target oviposit and feeding occurs. In the absence of the preferred host *C. nutans*, *R. conicus* fed on thistles with similar phenology and synchronous flowering times, which reduces seed set and population viability of the native thistle [32,34]. Prediction of non-target host selection for *R. conicus* would not have identified the native thistle *C. canescens* as a host plant based on plant morphology and the phylogenetic relationships (Figure 1). Likewise, prediction of non-target host selection for the foliar feeder *Trichosirocalus horridus*, which also was introduced from Europe into the U.S. in late 1960s as biological control agent for *C. nutans* [35], may not have identified the potential for foliar damage observed on North American native thistles *C. altissimum*, *C. discolor*, and *C. carolinianum* [36]. It is now known that various ecological factors like habitat preference of the biological control agent and geographical proximity to related plants provide better cues to potential alternative hosts [32,36,37,38].

Pathogens can be a particularly useful tool for weed control in natural areas that are rich in valued non-target species [39]. The fungal pathogen *P. carduorum* was evaluated as a biological control agent for *Carduus* spp. (musk thistles) [24]. *P. carduorum* collected from Turkey and Bulgaria was inoculated on three large flowered *Carduus* spp., twenty-four *Cirsium* spp., and *C. scolymus*. The *Cirsium* spp. selected for screening included a portion of the taxa that geographically overlapped the targeted *Carduus thoemeri*. In contrast with the above mentioned insect biological control agents, the strain of rust fungus tested by Bruckart *et al*. [24] coincides with plant host phylogenetic lines (Figure 1), as only *Carduus* spp. were susceptible. More extensive testing on seven rare, endangered, or threatened *Cirsium* spp. in California and extensive analysis using molecular marker data support that the rust strain only affected *Carduus* spp. [40,41].

*Cirsium* is a genus with a high affinity to form natural interspecific hybrids [42]. Fortunately, we did not detect interspecific hybridization between the introduced invasive and the native North American *Cirsium* spp. because such hybrids may provide a bridge for movement of host-specific biological control agents to expand their host range to non-target parental plants [43]. However, we did detect higher levels of variation within the invasive- relative to the native-*Cirsium* spp. This intraspecific genetic variation in the *C. arvense* may present challenges for identification of highly efficacious host-specific biological control agents. Molecular-based approaches that evaluate the phylogenetic or genetic diversity of invasive host plants and insect and pathogen biological control agents will be important for matching hosts and potential biological control agents [43]. Beyond the molecular-based pairing and the phylogenetic methods for delineating host range [44], it will be important to evaluate ecological factors to provide better cues to potential alternative hosts since some biological control agents do not follow host phylogenetic lines [37]. 

## 4. Experimental Section

### 4.1. Plant Material

Leaf material was collected from 15 to 43 individuals per population of *Cirsium arvense* (Table 1) and either dried with silica gel or kept at 4–8 °C until frozen at −80°C. Material from other *Cirsium* spp. (Table 1) was collected from 1 to 6 individuals per population and stored as described. Genomic DNA was extracted using the DNEasy kit (Qiagen Inc., Valencia, CA, USA). The DNA was quantified by spectrophotometry (Nanodrop Technologies, Wilmington, DE, USA).

Molecular markers for 26 samples representing eleven Great Plains *Cirsium*, two *Carduus*, and a *Cynara* (artichoke) species were sequenced to compile a matrix with published *Cirsium* sequences (Table 1). Multiple populations were included for species to examine variation between conspecifics. Populations with multiple species of *Cirsium* were included to determine if interspecific hybridization occurs between native and introduced species.

### 4.2. Amplification and Sequencing

Primers previously used for amplification of Cardueae (18S-ETS and ETS-Car1 and ITS1 and ITS4) [20] successfully amplified the regions for all samples. Reaction conditions were 1× Buffer E (Epicentre Biotechnologies, Madison, WI, USA), 0.5 mM primer, and 0.5 U Taq Polymerase (Promega Corp., Madison, WI, USA) with 10–25 ng genomic DNA. Amplification program parameters for ITS and ETS regions were those of Kelch and Baldwin [20]. Polymerase chain reaction (PCR) products were purified using a QiaQuick Gel Extraction kit (Qiagen Inc, Valencia, CA, USA) and quantified by spectrophotometery. Sequences were obtained using amplification primers (5 pmol) and 20–50 ng PCR product and sequenced with an ABI 3730 DNA Analyzer (Applied Biosystems, Inc., Foster City, CA, USA).

### 4.3. Data Analysis

Contiguous consensus sequences were compiled from double stranded DNA using Seqman (LaserGene, DNAStar, Madison, WI, USA). Alignments for the combined and independent data sets were produced in CLUSTALX, with gaps treated as missing data [45]. We deposited new DNA sequences in GenBank (Table 1). Phylogenetic analyses were conducted in PAUP*4.0b10 [46] with random stepwise addition of 100 iterations and tree bisection-reconnection branch-swapping. Partition homogeneity analysis in PAUP identified if the ITS and ETS were incongruent. The HKY85 model of evolution was used for maximum likelihood analysis [20]. Divergence between samples was calculated in PAUP as pairwise sequence similarity.

Several introduced and native biological control agents (e.g., insects and pathogens) were mapped onto the phylogeny to examine patterns of host preference [8,14,17,36,40]. The non-native, introduced insects were *L. planus*, *R. conicus*, and *Trichosirocalus horridus*, native pathogens were the rust pathogen P*uccinia carduorum*, and the bacterial pathogen *P. syringae* pv. *tagetis*.

## 5. Conclusions

From this research we conclude that there has not been interspecific hybridization between the introduced invasive such as Canada thistle and the native North American *Cirsium* spp. In addition, within *Cirsium*-Cardueae, the insect biological control agents do not associate with host phylogenetic lines. Thus, more comprehensive testing of species in host-specificity trials, rather than relying on a single representative of a given clade may be necessary; because the assumption that host-specificity follows phylogeny does not necessarily hold. Even if the assumption does not always hold, it is also important to evaluate ecological factors like habitat preference of the biological control agent and geographical proximity to related plants to provide better cues for host specificity [3,34].
